# Flexible Bandpass Filter Fabricated on Polyimide Substrate by Surface Modification and In Situ Self-Metallization Technique

**DOI:** 10.3390/polym11122068

**Published:** 2019-12-12

**Authors:** Huiwen Qu, Zhiliang Wang, Dingyong Cang

**Affiliations:** School of Information Science and Technology, Nantong University, NanTong 226019, China; quhw1721@163.com (H.Q.); cdingyong@163.com (D.C.)

**Keywords:** flexible electronics, polyimide substrate, bandpass filter, surface modification, in situ self-metallization

## Abstract

Polymer, especially polyimide (PI), is the best suitable substrate material for the design of flexible electronics. The compact silver can be reduced on the surface of PI films by surface modification and in situ self-metallization technique. The formed silver layers have good electrical and mechanical flexibility. A flexible bandpass filter on a PI flexible substrate by surface modification and in situ self-metallization technique at room temperature are presented in this work. Measured results show that the proposed flexible bandpass filter could achieve a fractional bandwidth of 80.8% with an insertion loss (IL) of less than 0.6 dB. The performance of the designed filter is almost constant under different bending, folding, and rolling conditions. The formed silver layers also present good adhesion with PI substrates. This technology provides an alternative approach for manufacturing flexible filters without high-temperature thermal annealing, costly equipment, and vacuum conditions.

## 1. Introduction

Polymers are suitable for use in the substrates of flexible electronics due to their excellent bendability, electrical properties, and other characteristics [1,2,3,4,5,6]. Among them, liquid crystal polymer (LCP) [1,2] and polyimide (PI) [3,4] have recently been widely used in substrates of flexible devices. PI is cheaper than LCP [7]. Flexible electronics, such as antennas [8,9], filters [7], and sensors [10], are typically fabricated on the PI substrate. 

The mainstream fabrication techniques for electronic applications on PI substrates consist of photolithography [11] and printing techniques (such as screen printing, inkjet printing, and gravure printing) [12,13,14]. However, these proposed approaches require stringent environmental control, costly equipment, and complex multistep processes [15]. In addition, it is difficult to obtain excellent adhesion on the very smooth PI substrate [16]. In order to improve the adhesion of passive metals on the PI substrate, surface modification and in situ self-metallization technique was presented [16,17]. This technique requires no high-temperature thermal annealing, costly equipment, or vacuum conditions [18]. In the previous work, antennas on PI substrates [18] were fabricated by the proposed technique, and the performance of the fabricated antenna was excellent, demonstrating that the proposed technique could be used to manufacture microwave devices. As a key component of the radio frequency (RF) front, bandpass filters should also be developed for flexible devices. However, to the author’s knowledge, no papers have been reported about the research on fabricating flexible bandpass filters by surface modification and in situ self-metallization technique. Therefore, designed filters are prepared by the proposed technique and measured to verify whether this technology can be used in the field of filters.

In this work, flexible bandpass filters fabricated on PI substrates using surface modification and in situ self-metallization technique at room temperature are presented. Under different bending, folding, and rolling conditions, the measured bandwidth and insertion loss of the flexible filter show good consistency. Meanwhile, the adhesion of passive metals to the substrate of the flexible filter was also tested, and the results showcase high reliability. The combined performance of the proposed flexible bandpass filter is outstanding for applications in flexible electronics.

## 2. Materials and Methods 

### 2.1. Design of the Filter

**Figure 1:**
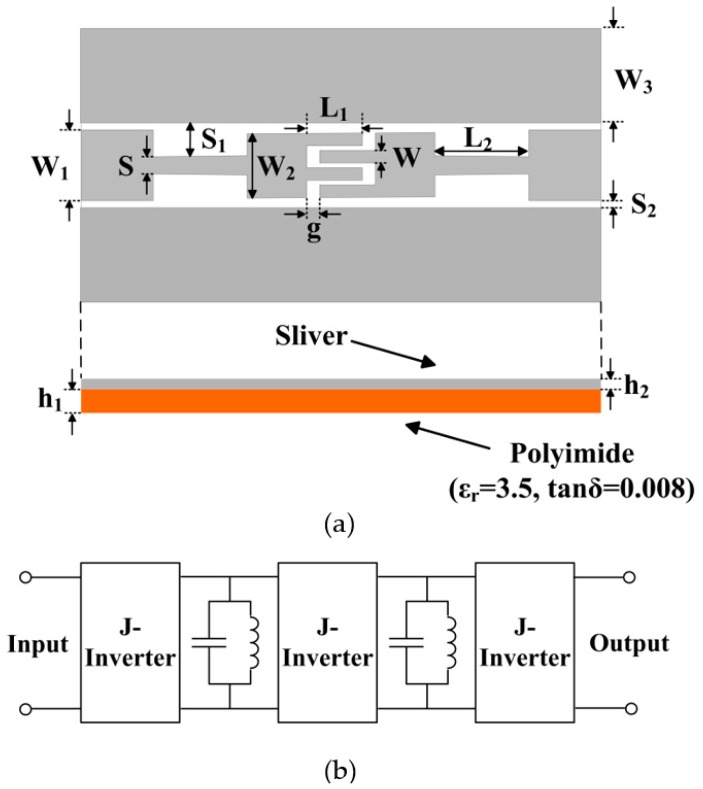
(**a**) Schematic view and (**b**) circuit diagram of the bandpass filter.

The flexible bandpass filter is designed on 50 μm PI substrate (ε_r_ = 3.5, tanδ = 0.008). Geometry and circuit of the two-stage bandpass filter are shown in Figure 1. Coplanar waveguide (CPW) and J-inverter structures are used to design the filter. The proposed bandpass filter uses the interdigital capacitor on center and high impedance inverter structures on two ends of the filter. In order to match the characteristic impedance, the input and output ports of the flexible bandpass filter are a 50 Ω CPW transmission line [19], where the strip and slot widths are 4.5 and 0.2 mm. respectively. All parameters were adjusted to acquire optimized filter performance. The final size of the bandpass filter was 20.9 mm × 43.1 mm, and the specific dimensions are as follows: W_1_ = 4.5, S_1_ = 0.9, S_2_ = 2, W_2_ = 4.1, g = 0.2, L_1_ = 4.7, W = 0.8, L_3_ = 4.2, S = 0.4, L_2_ = 8.8, W_3_ = 8, h_1_ = 0.05, h_2_ = 0.0076, all in millimeters.

### 2.2. Fabrication of the Filter

Commercial 50 μm PI films were produced from Qian Feng Insulating Material Plant (Shanghai, China). KOH, AgNO_3_, NH_3_·H_2_O, and H_2_O_2_ (30%) were purchased from Aladdin Industrial Corporation (Shanghai, China) and were used without further purification. 

**Figure 2:**
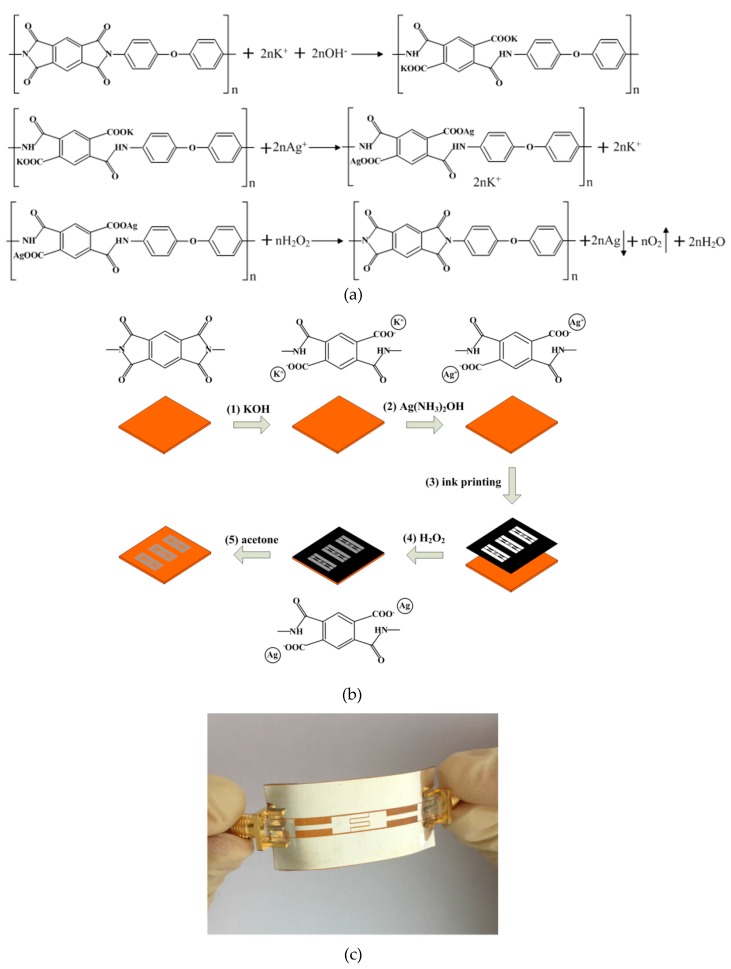
(**a**) The major chemical reactions of the silvered polyimide (PI) films. (**b**) Schematic illustration of the main steps and of the flexible filter. (**c**) Image of the flexible bandpass filter.

PI films are not resistant to hydrolysis under alkaline conditions [10]. As shown in Figure 2a, the preparation of the silvered PI films included steps of hydrolyzing, ion exchange, and reduction reaction. The surface of the PI films was modified by potassium hydroxide solution. Then, silver ammonia solution was used for ion exchange reaction. Finally, hydrogen peroxide was used for reduction treatment. 

The main steps of the flexible bandpass filter fabrication are shown in Figure 2b. This experiment was carried out at a room temperature of 25 °C. The surface modification was the first step. Then, the cleaned PI film was immersed in 4 M KOH solution for 3.5 h. After that, the PI surface was modified into poly(amic acid) (PAA). After being washed, the modified surface was immersed in 0.04 M Ag(NH_3_)_2_OH solution for 2 h, which resulted in the formation of silver ion-doped layers. Thereafter, the cleaned and dried film was stuck flatly on paper. Afterwards, the outer area of the designed bandpass filter structure was printed on the modified PI film. Subsequently, the printed film was dipped into 0.1 M H_2_O_2_ (30%) solution for ten seconds. The adsorbed silver ions were reduced to metallic silver. Finally, the masking pattern was removed by using acetone solution, and the proposed bandpass filter was prepared. The specific fabrication procedure for manufacturing the silvered PI film can be found in the literature [8,16].

Figure 2c shows a photo of the flexible bandpass filter on the PI film. The two subminiature version A (SMA) connectors were used to connect the flexible bandpass filter for tests. It can be seen that no crack or contamination occurred during the reduction process. The designed bandpass filter has a great potential to be integrated with other flexible electronic components [20]. This technique does not rely on costly equipment, and the adhesion of passive metals to the polymers is better.

## 3. Results

### 3.1. Crystal Structures

The prepared slivered PI film was investigated by X-ray diffraction (XRD, Ultima IV, Rigaku, Tokyo, Japan). The scanning angle was between 30° and 80°, the step size was 0.02°, and the scanning speed was 5°/min.

**Figure 3:**
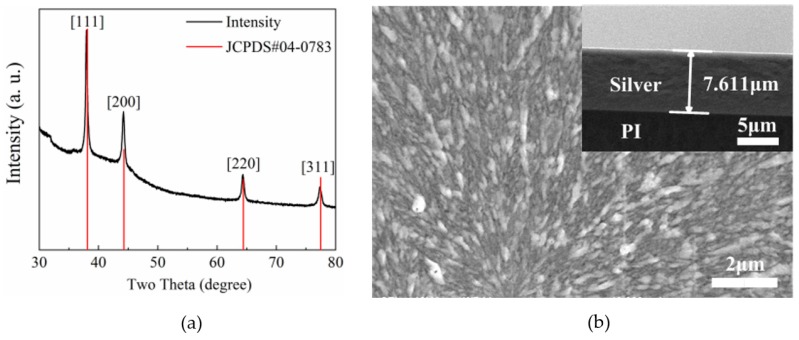
(**a**) The X-ray diffraction (XRD) image and (**b**) top and cross-sectional scanning electron microscopy (SEM) images of the silvered PI film.

As shown in Figure 3a, the XRD patterns tally well with the data of the standard JCPDS (04-0783), indicating that the silvered PI film surface is constructed by face-centered cubic silver crystalline particles [18].

### 3.2. Morphologies

Surfaces and cross-section images of the prepared composite membrane were identified by scanning electron microscopy (SEM) (JSM-6510, JEOL Ltd., Tokyo, Japan).

As shown in Figure 3b, the surface morphology shows that uniform and dense silver layers have been obtained. The insertion in Figure 3b indicates that the thickness of the formed metallized layer is approximately 7.611 μm. According to the literature [10,17,18], it is found that when the PI film was immersed in 4 M KOH solution for 3.5 h and 0.04 M Ag(NH_3_)_2_OH solution for 2 h, a silver layer with a thickness of about 7.6 μm can be obtained. At this time, the silver layer has the smallest resistivity, which meets the requirements for the filters.

### 3.3. Characteristics

The transmission (S_21_) and reflection (S_11_) characteristics of the flexible bandpass filter are measured by using a Vector Network Analyzer (VNA, Agilent E8363C, Agilent Technologies Inc., Santa Clara, CA, USA).

**Figure 4:**
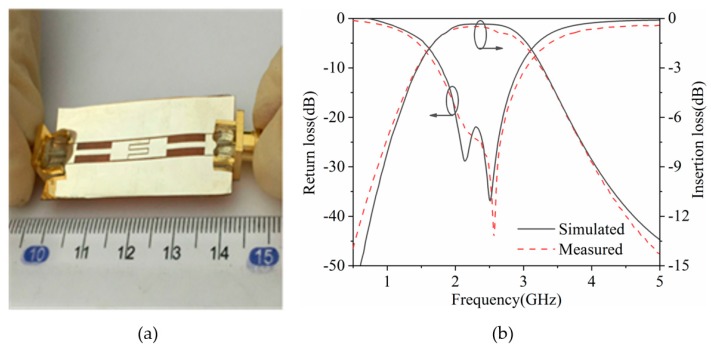
(**a**) Photograph and (**b**) S-parameters of the flexible filter in a flat condition.

The photo of the filter in a flat condition is shown in Figure 4a. As shown in Figure 4b, the simulated and measured curves of the designed flexible bandpass filter are plotted and compared in a flat condition. The simulated results show that the flexible bandpass filter had a center frequency of 2.42 GHz, a 3 dB bandwidth of 1.76 GHz, a fractional bandwidth of 73.2%, a minimum insertion loss of 0.34 dB, and a return loss of more than 20 dB. The measured results of the filter show that the relative 3 dB bandwidth was 1.94 GHz, its center frequency was 2.42 GHz, and the relative bandwidth was 80.8%. The minimum insertion loss of the measured prototype was 0.49 dB. The measured results are basically consistent with the simulation and meet the design requirements. The errors between simulated and measured results are mainly caused by the influence of the testing environment and the fabrication tolerance [20,21]. 

To investigate the mechanical flexibility of the flexible bandpass filter, the performance of the filter was tested under different conditions such as bending, folding, and rolling.

**Figure 5:**
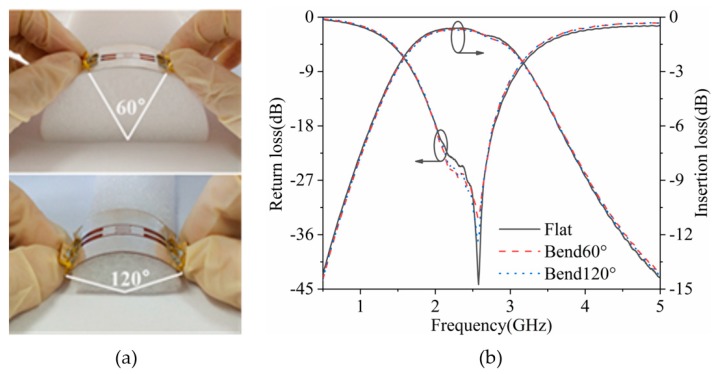
(**a**) Photographs and (**b**) S-parameters of the flexible filter under different bending conditions.

Figure 5 shows the photos and performance of the flexible filter when it is bent 60° and 120°, respectively. Compared with the flat case, its relative 3 dB bandwidth, center frequency, and minimum insertion loss are similar. Although the maximum return loss has experienced different degrees of deterioration, it still meets the basic requirements of the filter design. Therefore, the proposed filter still has good performance after being bent.

**Figure 6:**
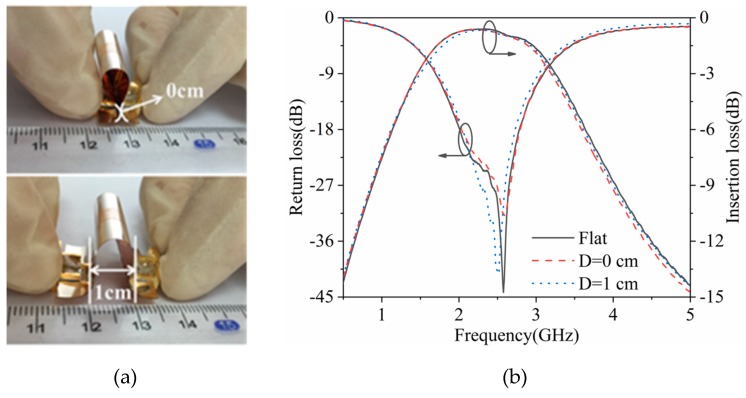
(**a**) Photograph and (**b**) S-parameters of the flexible filter under different folding conditions.

As can be seen in Figure 6, the flexible filter was put under different folding conditions, and the distances of the two connectors were either 0 or 1 cm. Compared with a flat condition, in the case of D = 0 and 1 cm, the maximum return loss deteriorated by 12.29 and 2.90 dB, respectively, and the minimum insertion loss deteriorated by 0.02 and 0.02 dB, respectively. Other than these, the center frequency and the bandwidth were basically unchanged. There was a very small susceptibility to folding.

**Figure 7:**
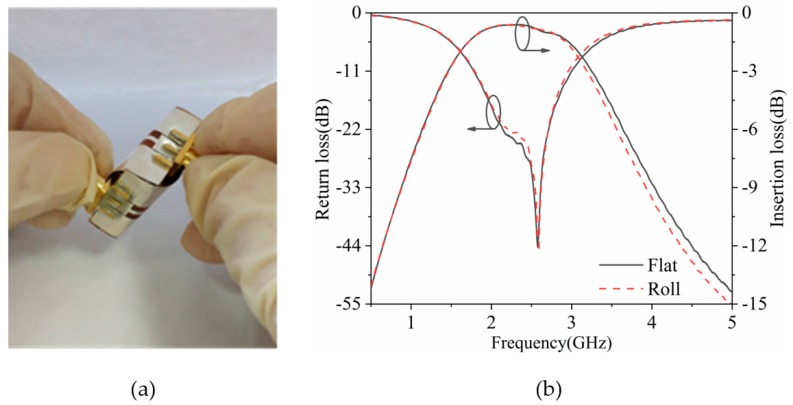
(**a**) Photograph and (**b**) S-parameters of the flexible filter under a rolling condition.

As shown in Figure 7a, the fabricated bandpass filter was measured under a rolling state. Figure 7b indicates that the measured minimum insertion loss deteriorated by 0.1 dB and the maximum return loss improved by 0.2 dB. These results indicate that the characteristics of the proposed bandpass filter are well preserved.

**Figure 8:**
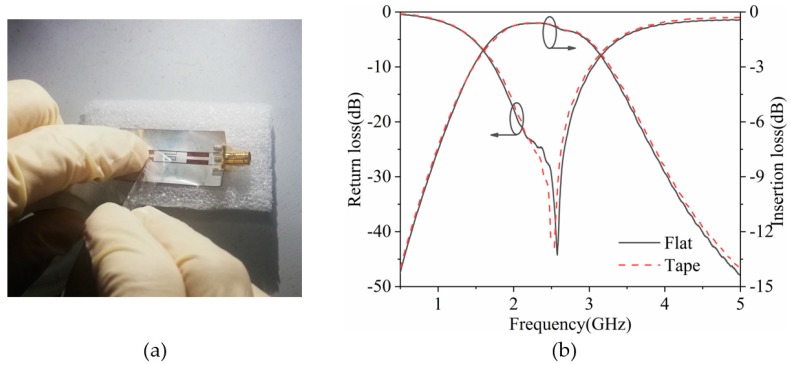
(**a**) Photograph and (**b**) S-parameters of the flexible filter after being taped.

To demonstrate the adhesion of passive metals to PI films, the designed flexible filter was submitted to an adhesive test by using normal tape. Figure 8 shows the measured curves of the filter under the flat state after 100 test cycles. The S-parameters of the filter were similar, proving the durability of the flexible filter in the experiments.

The flexible filters fabricated by other techniques in the literature [22,23,24] were usually only tested when the filter was bent. The flexible filters manufactured by surface modification and in situ self-metallization technology were tested under bending, folding, and rolling conditions. As seen in Table 1, the minimum insertion loss of the bandpass filter deteriorated by 0.01, 0.02, 0.02, and 0.02 dB separately after the bending, folding, rolling, and tape-exfoliation tests. The errors were mainly caused by the influence of testing environments and fabrication tolerance [20,21]. The frequency shift and the insertion loss degradation of the designed filters were negligible.

## 4. Conclusions

In summary, a flexible bandpass filter was fabricated on a 50 μm PI film by surface modification and in situ self-metallization technique. This technique does not rely on costly equipment and the adhesion of passive metals to polymers is better. When the flexible bandpass filter is in a flat state, measured results show a center frequency of 2.42 GHz, a 3 dB bandwidth of 1.92 GHz, a minimum insertion loss of 0.6 dB, an insertion loss of more than 20 dB, and a relative bandwidth of 80.8%. The electrical performance of the flexible bandpass filter remained almost unchanged after being bent, folded, and rolled. The high mechanical flexibility of the designed filters indicates that the surface modification of the film and in situ self-metallization technique provides a new preparation method for the polymer and expands the application field.

## Figures and Tables

**Table 1:** Comparison of fabrication methods for flexible bandpass filters.

Fabrication Method	Center Frequency (f_0_) (GHz)					Minimum Insertion Loss (|S_21_|) (dB)					Ref.
	Flat	Bend	Fold	Roll	Tape	Flat	Bend	Fold	Roll	Tape	
Printing	7.35	7.20(45) ^1^	NA ^2^	NA	NA	0.80	0.80(45)	NA	NA	NA	[22]
	5.53	5.61(37)	NA	NA	NA	1.9	2.03(37)	NA	NA	NA	[23]
Photolithography	2.48	2.48(15)	NA	NA	NA	1.59	1.59(15)	NA	NA	NA	[24]
In this work	2.42	2.42(60)	2.42	2.42	2.42	0.49	0.50(60)	0.51	0.51	0.51	

^1^ The angle when the filter is bent; ^2^ NA = not available.
